# High resolution behavioral and neural activity representation using a geometrical approach

**DOI:** 10.1038/s41598-020-64726-6

**Published:** 2020-05-14

**Authors:** Zev Brand, Avi Avital

**Affiliations:** Behavioral Neuroscience lab, Gutwirth Building, Department of Neuroscience, Faculty of Medicine and Emek Medical Center, Technion - Israel Institute of Technology, Haifa, 32000, Israel

**Keywords:** Psychophysics, Attention

## Abstract

Available tools for recording neuronal activity are limited and reductive due to massive data arising from high-frequency measurements. We have developed a method that utilizes variance within the physiological activity and includes all data points per measurement. Data is expressed geometrically in a physiologically meaningful manner, to represent a precise and detailed view of the recorded neural activity. The recorded raw data from any pair of electrodes was plotted and following a covariance calculation, an eigenvalues and chi-square distribution were used to define the ellipse which bounds 95% of the raw data. We validated our method by correlating specific behavioral observation and physiological activity with behavioral tasks that require similar body movement but potentially involve significantly different neuronal activity. Graphical representation of telemetrically recorded data generates a scatter plot with distinct elliptic geometrical properties that clearly and significantly correlated with behavior. Our reproducible approach improves on existing methods by allowing a dynamic, accurate and comprehensive correlate using an intuitive output. Long-term, it may serve as the basis for advanced machine learning applications and animal-based artificial intelligence models aimed at predicting or characterizing behavior.

## Introduction

Available tools for recording neural activity are limited by the large amount of data arising from high frequency (20 kHz) measurements. As a result, current approaches are bound to be reductive in essence and capture mostly significant, robust events within the data (e.g. spikes), most likely losing resolution, or neglecting potentially important events. Traditionally, a single-channel analog signal is separately recorded and subsequently processed and analyzed^1,2^. Recent advancements include a multichannel system, where analog-to-digital signals are done on the animal’s head stage. The digital signal is then relayed using radiofrequency for further analysis (e.g. spike sorting). There are few methods to display a group of neurons starting from the classic spike raster plot which displays only the spiking of each neuron and not the whole activity^3,4^. Additionally, decoding the raster plot to depict synchronized spiking and the quantification of the synchrony level are not intuitive nor measurable. The peristimulus time histograms (PSTHs) method has better resolution by relating to the firing rate of each neuron^3^. However, it doesn’t offer better quantification or synchrony of all the recorded neurons. Most recent studies by Doya and others, have dramatically improved the way we capture and analyze the signal by using spectrograms that are created based on high/low frequency^5,6^. Nevertheless, the spectrogram approach is representing each neuron separately and thus missing the inter-neuron-correlation. Another limitation of current approaches is the lack of live synchronization - the ability to synchronize the entirely recorded neural activity with behavior as it occurs. To achieve that, data collection needs to be inclusive and there should be a method that allows such comprehensive recording and analysis, that is also physiologically meaningful.

In the current study, we have developed a method that overcomes computational challenges encountered by current approaches. This method avoids data filtration or spike sorting, comprises all data points for each measurement and relies on the variance observed within the physiological activity. Data are then expressed geometrically in a statistically- and physiologically-meaningful manner to depict a precise, detailed and holistic view of the recorded neural activity. We validated our method by investigating the neuronal striatal mechanisms of attention.

The striatum is known to manage executive functions by integrating sensorimotor, emotional and cognitive information; mainly action selection^7^. It has been reported that striatal malfunctioning impaired the ability to filter stimuli, therefore, the striatal pre-attentive role appears to be highly important^8^. Nevertheless, the intra-striatal mechanisms that govern the pre-attentive behaviors are yet to be investigated. Two brain regions, the Motor Cortex and the Putamen (resides in the striatum) are reported to be involved in motor function and attention-induced decision making^9,10^. Together, the main aim of our study is to seek the correlation between these two regions while performing ‘low’ vs. ‘high’ selective attention tasks.

### Results neural activity analysis and characterization

**Figure 1:**
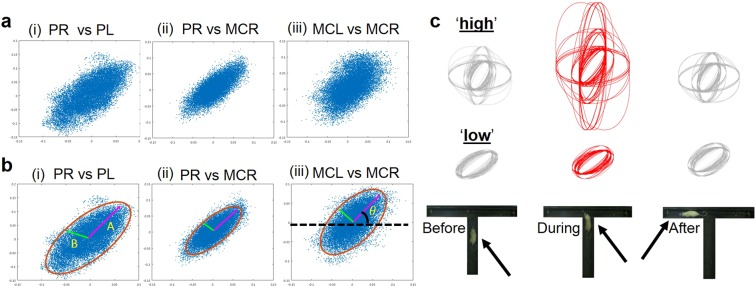
Neural activity recording and analysis. (**a**) Scattered plots of 1 second of neural activity recording. The x- and y- axes display a representative recorded raw data in millivolts coming from the following electrode combinations: i) Putamen right (PR) vs Putamen left (PL); ii) PR vs Motor cortex right (MCR); iii) MCR vs Motor cortex left (MCL). (**b**) Statistical and geometrical characterization of recoreded data. Ellipse shape with 95% confidence level is represented by the red line. In magenta, ellipse major axis A; in green elipse minor axis B. i) PR vs PL; ii) PR vs MCR; iii) MCR vs MCL. (**c**) A comparison of data recorded from eight electrodes located at the PR and the PL, yielding 64 ellipses for each phase of the task (before,during and after) in ‘high’ or ‘low’ attention conditions.

We examined the possible association between the neural activity recorded from any pair of electrodes within the Putamen and the Motor cortex, pustulating they reflect both reward processing and the motor actuation, respectively^11^. To achieve that, we plotted the raw data recordings coming from any two linear multielectrode (eight contacts in each multielectrode). Specifically, while recording in 20 kHz, we plotted the raw data scatter of any two contacts coming from two out of four different regions (right/left Motor cortex or right/left Putamen) (Fig. 1a). Examining 64 scatter plots for every second of recording during the abovementioned behavioral phases, we observed an elliptic-like dynamics for any pair of electrodes from all regions we plotted. We further characterized the geometrical properties of the observed ellipse-like shapes. We calculated the covariance matrix of the raw data from each pair of electrodes, second-by-second and found the Eigen values and vectors of the covariance matrixes. The Eigen values represent the variance of the data in the direction of the Eigen vectors. The arc tangent of the x and y axes of the ellipse’s larger Eigen vector is considered as the ellipse orientation (see online methods for detailed explanation and an example). We used the chi-square distribution table to meet a 95% confidence interval and multiplied the Eigen values by the square root of 5.99. The outcome was the ellipse’ various parameters (Fig. 1b).

### Synchronization of neural activity with behavioral performance

We have investigated a possible functional correlation between neural activity and behavioral performance by synchronizing the geometrical features of the ellipses with the behavioral performance of the rats. To achieve that, we produced an ellipse shape for every second during the behavioral trial. Behavioral performance was divided into three phases: *before*, *during* and *after* turning from the main arm into the perpendicular arm(s). For any two sub-regions, we calculated the covariance between 8 × 8 of electrodes, yielding 64 ellipses at each time point (Fig. 1c). We quantified the differences between the ellipses along the *before, during* and *after* phases of the ‘low’ versus ‘high’ attention conditions. Per each ellipse, we characterized A and B axes, Theta – ellipse orientation, X and Y projections, and the ellipse area.

### Validation of the geometrical approach

We examined whether any of the geometrical features consistently and significantly reflected the electrophysiological recordings as a function of the rat’s behavior. We measured the two selective attention conditions (‘low’ vs. ‘high’) in three different phases of the activity (*before*, *during* and *after*) per each. Initial statistical analyses (using Two-way ANOVA followed by Post-hoc Tukey tests) of the contralateral signals of the putamen (Right putamen (PR) vs left putamen (PL)) revealed that all geometrical features significantly discriminated between the *before* - *during* and the *during* - *after* phases, but not between the *before* - *after* phases within each attention condition. Interestingly, at this point we could not discriminate between ‘high’ and ‘low’ attention conditions using our geometrical approach (Table 1).

**Table 1:** Comparison of geometrical features of contralateral electrophysiological signals (PR vs PL) in different phases of the task (*before, during, after)*.

Phases	Geometrical features					
	A	B	THETA	X	Y	AREA
Before vs During	*P* < 0.0001	*P* < 0.0001	*P* < 0.0001	*P* < 0.0001	*P* < 0.0001	*P* < 0.0001
Before vs After	*P* > 0.135	*P* > 0.455	*P* > 0.185	*P* > 0.537	*P* > 0.834	*P* > 0.963
After vs During	*P* < 0.0001	*P* < 0.0001	*P* < 0.0001	*P* < 0.0001	*P* < 0.0001	*P* < 0.0001

A statistical analysis *(*Two-way ANOVA followed by Post-hoc Tukey test; Sig. level <0.05) of the differences observed in the geometrical representation of the electrophysiological signals in different phases of a task. Each row represents a comparison between 2 different phases within the activity.

**Figure 2:**
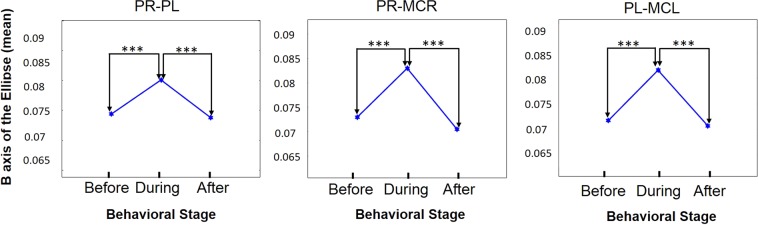
The B geometrical feature significantly discriminates between the *before-during* and the *during-after* phases of the task, regardless of ‘high’ vs ‘low’ attention conditions. A graphical representation of the means of the B feature in the these phases of the task. Putamen right (PR); Putamen left (PL); Motor cortex (MCR) right; Motor cortex left (MCL). One-way ANOVA followed by Tukey test revealed a consistent difference between the during phase and the before/after phases (*** *P*<0.0001).

Post-hoc Tukey test was used to investigate whether the B feature can differentiate between the three phases within a task (Fig. 2). In agreement with our initial findings, the B geometrical feature of the ellipses reflected the behavioral differences observed in the maze. We found a consistent and statistically significant difference in the B feature when we compared the *before*-*during* and *after*-*during* phases regardless of ‘high’ and ‘low’ attention conditions (*P* < 0.0001 for all comparisons of any two brain regions) and when comparing any two contralateral or ipsilateral examined brain regions. Consistently with our initial observation (Table 1), there were no statistically significant differences between the *before*-*after* phases (Fig. 2; Supp Table 1).

**Figure 3:**
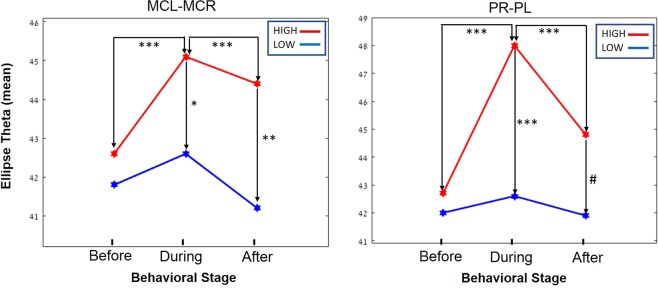
The Theta geometrical feature significantly discriminates between the high vs low attention condition and the *before-during* and the *during-after* phases of a task when recording contralaterally. A graphical representation of the marginal means of the Theta feature in the ‘high’ (red) and ‘low’ (blue) attention conditions during the three phases of the task. PR, Putamen right; PL, Putamen left; MCR, Motor cortex right; MCL, Motor cortex left. (Tukey tests: ***P < 0.0001; **P < 0.004; *P < 0.009; ^#^*P* < 0.01).

We investigated whether there is a specific geometrical feature that discriminates between the ‘low’ vs. ‘high’ attention conditions. We found that the Theta geometrical (the ellipse’s rotation angel) feature successfully discriminated between those two attention conditions. This also explains the rotary presentation observed in the *during* phase of the ‘high’-attention task. Additionally, the Theta feature discriminated between the *before-during* and the *during-after* phases when comparing contralateral regions of the brain (e.g., PL vs PR) (Fig. 3).

As can be observed in Fig. 3, the Theta value for the *after* phase in the ‘high’ attention condition was significantly higher than that of the *after* phase in the ‘low’ attention task (Fig. 1c illustrates these findings). This can be explained by our observation that the rat is aware there is also food in the familiar ‘low’ attention arm. Indeed, in the ‘high’ attention trials, after consuming the food in the new arm, the rat went back to the familiar arm 100% of the times and consumed the food that was there.

## Discussion

Our newly developed method uses geometrical analysis of neural activity to successfully discriminate between the contingent different behaviors and examine them in a meaningful, reproducible manner.

Our approach is unique as, in contrast to other available methods, it uses all, unfiltered recorded neural signals. Current methods such as raster plot^3,4^, PTSH^3^ and frequency transformation spectrograms^12^ share a lack of synchronization between all recorded neurons. To that end, we synchronized all recorded neurons (20 kHz) from different brain areas and different phases of the behavioral conditions. Comparing any two brain areas for any given examined time point produced an ellipse-shaped scatter plot with distinct geometrical features that consistently and differentially represent electrophysiology and behavior synchronization. We have validated the use of this approach, comparing ‘low’ and ‘high’ attention conditions. For each condition, we investigated behavior in distinct phases of activity and successfully attributed geometrical features to different behaviors. We found that when we compare the contralateral signals of the putamen (PL-PR), all geometrical features discriminated between the different phases of a given behavior but did not discriminate between ‘high’ versus ‘low’ attention behaviors. The B parameter reliably discriminated between the two attention conditions and the three phases within each task. It is important to note however, that the interaction was not ordinal. This may reflect a possible interchangeable equilibrium between the recordings that follows either the attention condition or the performance phase. Supportive of this is the consistency of the results from both ipsilateral and contralateral signals (e.g., PR-MR, ML-PL). Furthermore, the Theta feature distinctively discriminated between the ‘high’ and ‘low’ attention conditions and the different phases within the task when we compared contralateral regions. We noted that in ‘low’ attention condition, the Theta value in the *during* phase was lower than its value in ‘high’ attention condition, which means a smaller rotation of the ellipse. We postulate that the rotation indicated by Theta reflects the modulated equilibrium of the neural activity between each pair of electrodes from the two brain regions. This change in equilibrium involves relevant brain regions: the putamen for attentional processing and the motor cortex as an executer. The contralateral effect as well as the specific rotation effect reflected by the Theta feature may be viewed as a functional reconfiguration of the neural networks. This notion is supported by Sauvage *et al.*^13^ who showed that a differential and coupled recruitment of cognitive networks can constitute a neural marker of training effects, based on shift of activity between brain regions. Specifically, we suggest that the activity shift represented geometrically by Theta reflects the shift from ipsi- to contra-lateral when there is a need for increased attention and information integration. In our model, those are characteristics of the *during* phase.

In conclusion, our newly developed and validated method represents an improved and reproducible way to neurally represent behavior. Our method is not reductive but rather intuitive and with validated physiological relevance. It can be expanded and investigated for any neurophysiological research that uses neural recording at high temporal and spatial resolution imaging aimed also to be functionally correlated with behavior. A limitation of our method is the need to acquire tremendous number of neural recordings with synchronization to a well-defined behavioral task. More research, in complex learning settings, is required to generalize our method’s reproducibility. Specifically, our method is limited by the restriction of examining two regions each time, and thus undermining the complexity of neural representation of behavior.

Our method summarizes significant amount of neural data into an ellipse’s geometrical features. Due to its high reproducibility, unbiased collection of data points and allowing the expression of behavior-physiological variance, the method may serve as the basis for advanced machine learning applications and animal-based artificial intelligence models aimed at predicting or characterizing behavior. Moreover, the clinical translation-related aspects of our research may be viewed in two levels: First, to give rise to the understanding of major psychopathologies (e.g. major depression, schizophrenia), our method can be implemented while examining animal models to better clarify the hypothesized dysregulated equilibrium of neural activity of relevant brain regions. Secondly, referring to human subjects, Electroencephalogram (EEG) that is commonly used, may also implement our geometrical representation.

In sum, our suggested method may serve to better understand the concurrent of covert neural activity and overt behavior, in both health and disease.

## Materials and Methods

### Animals

Five male Wistar rats (weighing between 300–350 g) at the age of 3 months were purchased from Harlan laboratories (Jerusalem, Israel) and reared in the institutional animal housing facility (Technion, Israel). Rats were housed 2 per cage (30 L × 30 W × 18 H cm) in a 23 ± 1 °C room temperature and ~67% humidity and acclimated to the housing facility for one week before experiments were started. A 12:12 day/night cycle (lights on at 6:00 am) and ad-libitum access to water were kept, and all manipulations and behavioral testing were held between 7:00 am and 5:00 pm.

All experimental procedures and protocols were approved by the Technion’s Institutional Animal Care and Use Committee (IL-028-02-2015; February 2015). All methods were carried out in accordance with the recommendations of the Guide for the Care and Use of Laboratory Animals of the National Institutes of Health, and all efforts made to minimize animal suffering. All five rats that were approved for the experiments participated with no special medical conditions nor mortality during the experiments.

### Electrode array implantation

Rats were anesthetized using Ketamine (90 mg/ kg) - Xylazine (10 mg/ kg), lidocaine (2%) was administered 20 minutes before a sagittal incision toward the skull and fur was removed using clippers. Rats were restrained in a stereotactic apparatus (Stoelting, IL, USA) and their body temperature of 37 °C was maintained using a heating pad. Bi-lateral opening was made in the skull with a trephine and microelectrodes were lowered through the Dura-matter and the brain at a rate of 1 mm per minute in order to minimize tissue damage.

Simultaneous bi-lateral recording was conducted from the Putamen and the Motor cortex, using a custom-made multi-layer linear electrode array comprised of 32 single unit recordings (i.e. 10 μm contacts).

The electrode array is lowered to the following coordinates: MC: AP: +1.08 mm, LM: ±2.2 mm, VD: −4.6 mm (see Fig. 1a). Dental cement was used to fix the electrodes on the head of the rat. Three stainless screws, each with a diameter of 1 mm were used to keep the installation fixed.

Rats were treated subcutanously with Buprenorphine (0.03 mg/kg) for two consecutive days for pain relief and were allowed 1 week to recuperate before being subjected to experimental procedures.

### Neural recording

We used a custom-made multilayer linear microelectrode array comprised of 32 Platinum/Iridium 12.5 μm diameter for single unit recordings, equally distributed between the two regions in both hemispheres, 100 μm apart (Alpha Omega, Israel, LTD). The multichannel communication system (Multichannel Systems Germany, LTD) transforms analog to digital signals on the head stage and sends the data (20 KHz) via radiofrequency (at 2.4 GHz) to the main computer, that telemetrically records it using the multichannel experimenter software.

### Behavioral task: ‘low’ vs. ‘high’ selective attention conditions

Examining a behavior in distinct phases is common in studies of selective attention and specifically in object recognition studies^14^.

**Figure 4:**
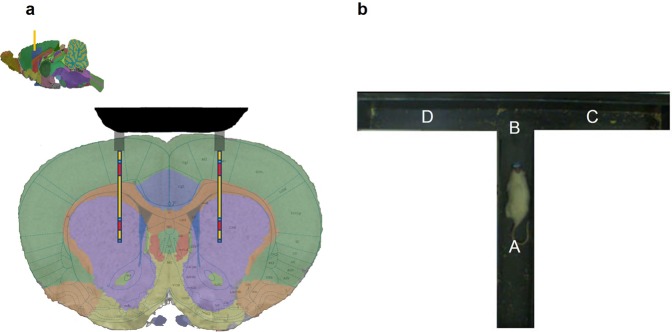
Experimental Setting. (**a**) A multi-layer electrode array located at: AP: +1.08 mm, LM: ± 2.2 mm, VD: −4.6 mm. (**b**) T-Maze setting. The rat is placed into the main arm (A) of the maze and the video recording begins by Ethovision. Next, Ethovision detects entrance to B zone and activates the Multichannel system via a costume-made device, to start neural recording. Neural recording system and video are still active as the rat turns into one of the two arms of the maze. They remain active until the rat receives its food reward and exists the C or D zones.

Following the implantation of microelectrodes bilaterally into the Motor Cortex and the Putamen (Fig. 4a), we have used a T-maze and selected a relatively simple behavioral task with either ‘low’ or ‘high’ attention conditions. While both conditions require similar body movements (which was the rationale for using a T-maze rather than an open box), they are assumed to involve dramatically different neural activity related to selective attention (Fig. 4b). A ‘low’ attention condition is defined as when the rat has only one turning option available (from the main arm to either zone C or D, see Fig. 4b) in the T-maze while the other arm is blocked. Rats were randomly assigned to right or left arms. The rat turning to the target arm is rewarded with 100 mg of cornflake. Rats are trained to perform the ‘low’ attention task for five consecutive days (6 trials per day). Thereafter, ‘high’ attention condition is introduced: the blockage of the second arm is removed which motivates (i.e. novelty exploration) the rat to pay attention to the additional and unfamiliar arm and choose between the familiar versus unfamiliar arms. Both arms were equally baited to exclude appetitive motivational imbalance. We have recorded the neural activity in both ‘low’ and ‘high’ attention conditions and in synchronization with three behavioral phases: (i) before a turn is made into the target arm; (ii) during the turn into the target; and (iii) after the decision has been made to choose the *familiar* or *unfamiliar* arm. All experiments were videotaped throughout the task, which allowed us to synchronize the behavioral performance with neural activity recordings at any chosen timepoint (Supplementary Video 1). The trials were videotaped with a color CCTV (WV-CP500(Panasonic camera, with an on-line analysis utilizing the Ethovision XT 9.0 software (Nuldus, The Netherlands). Our interfacing costume-made device synchronized the video tracking (i.e. entring to zone B – *during* phase) and activates the Multichannel, to start neural recording, using the multichannel communication and recording system.

### Geometrical representation analysis

We plotted the raw data recordings coming from any pair of electrodes (Fig. 1b) In the plots, the x- and y- axes are displaying the recorded raw data in pico-volts recorded from any pair of electrodes.

An axis-aligned ellipse, centered at the origin, with major axis of length 2a and a minor axis of length 2b, is defined by the following equation:

1 $${\left(\frac{x}{a}\right)}^{2}+{\left(\frac{y}{b}\right)}^{2}=s$$

The length of the axes is defined by the standard deviations ${\sigma }_{x}$ and ${\sigma }_{y}$ of the data such that the equation of the ellipse becomes:

2 $${\left(\frac{x}{{\sigma }_{x}}\right)}^{2}+{\left(\frac{y}{{\sigma }_{y}}\right)}^{2}=s$$

Because x-values (electrode 1) and the y-values (electrode 2) are normally distributed, the left hand side of equation (2) actually represents the sum of squares of independent normally distributed data samples. The sum of squared Gaussian data points is distributed according to a Chi-Square distribution. A Chi-Square distribution is defined in terms of ‘degrees of freedom’, which represents the number of unknowns. In our case there are two unknowns, and therefore two degrees of freedom.

Since we calculated the confidence interval, we looked for the probability that is less than or equals to a specific value which can be obtained using the cumulative Chi-Square distribution.

For example, using this probability table we can find that in the 2-degrees of freedom case:

3 $$P(s < 5.991)=1-0.05=0.95$$

Therefore, a 95% confidence interval corresponds to s=5.991. In other words, 95% of the data will fall inside the ellipse defined as:

4 $${\left(\frac{x}{{\sigma }_{x}}\right)}^{2}+{\left(\frac{y}{{\sigma }_{y}}\right)}^{2}=5.991$$

When the ellipse is not axis-aligned, we need to find the directions in which the 2D data has the largest variance. The directions in which the data varies the most are defined by the covariance matrix. It is known that the direction of the vectors along such a linear transformation are the eigenvectors of the transformation matrix. Indeed, the vectors shown by pink and green arrows in Fig. 1b, are the eigenvectors of the covariance matrix of the data, whereas the length of the vectors corresponds to the eigenvalues.

To obtain the orientation of the ellipse, we calculated the angle of the largest eigenvector towards the x-axis:

5 $$\theta =arctan\frac{{V}_{1}(y)}{{V}_{1}(x)}$$

where ${V}_{1}$ is the eigenvector of the covariance matrix that corresponds to the largest eigenvalue.

The major and minor ellipse are as follows:

6 $$a=\sqrt{{\chi }^{2}}\ast \sqrt{largest\_eigen\_val}$$

7 $$b=\sqrt{{\chi }^{2}}\ast \sqrt{smallest\_eigen\_val}$$

In order to characterize the repetitive ellipse shapes, we calculated the covariance matrix of the raw data coming from every pair of electrodes. The covariance of two random variables x and y is calculated by:

$$\sigma (x,y)=\frac{1}{n-1}\mathop{\sum }\limits_{i=1}^{n}({x}_{i}-\bar{x})({y}_{i}-\bar{y})$$

where x is a one electrode voltage array containing 20k samples(n) and y is the same for the second electrode. ${x}_{i}$ and ${y}_{i}\,$are the I’th voltage reading from each electrode, $\bar{x}$ and .. are the mean voltage for each electrode for that second.

since we are correlating two random variables our covariance matrix is two dimensional and is expressed by:

$$C=(\begin{array}{cc}\sigma (x,x) & \sigma (x,y)\\ \sigma (y,x) & \sigma (y,y)\end{array})$$

We found the Eigen values and vectors of the covariance matrixes. The Eigen values represent the variance of the data in the direction of the Eigen vectors. The arc tan of the x and y axes of the big Eigen vector is the orientation of the ellipse. Using the chi square confidence table, in order to meet 95% confidence level, we multiplied the Eigen values by the square root of 5.99. The outcome was the major and minor ellipse axes (Fig. 1b). In blue is a scatter plot of the recorded raw data, in red the ellipse shape with 95% confidence. In magenta is the ellipse major axis and in green the minor axis

In order to be able to synchronize the shape with behavioral performance, we produced an ellipse shape for every second of the behavioral trial.

Collapsing a series of ellipses along a full behavioral trial, we got a pattern of ellipses; every ellipse represents the covariance between a pair of electrodes (the example in Supplementary Fig. 1a depicts 6 ellipses of 6 seconds of recording each ellipse represents the covariance between the left vs. right Putamen). In order to compare the neural activity between two sub-regions, we produced a series of ellipses. The example in Supplementary Fig. 1b, depicts a bi-lateral comparison of 8 electrodes from the Putamen. Overall, we calculated the covariance between 8×8 of electrodes along 6 seconds, yielding 64 ellipses at each time point.

Considering the behavioral nature of the selective attention task, we chose a time sensitivity of 1 sec bins. However, the time sensitivity can be adjusted to sub-seconds sensitivity, depending on the behavioral task.

### Statistical analysis

Data was analyzed using one-way ANOVA followed by Post-hoc Tukey tests to compare the task phases (i.e. before, during and after). When examining both task phases and attention conditions (i.e. high or low), a two-way ANOVA (2 × 3) followed by Post-hoc Tukey was utilized to compare differences between the task phases. A student’s t-test was used as a Post-hoc when comparing two attention conditions. The statistical analyses were two-tailed and carried out by using SPSS (IBM, ltd; version 21). Results were considered significant when the *P*-value was less than 0.05. Results are displayed as mean ± S.E.M.

## Supplementary information

Supplementary information.

Supplementary Video 1.
