# Supplementary material for: High resolution behavioral and neural activity representation using a geometrical approach
Source: Sci Rep. 2020 May 14;10:7977. doi: 10.1038/s41598-020-64726-6 (PMC7224390; doi:10.1038/s41598-020-64726-6)
Supplement: Supplementary file 1 — Supplementary information. [file 41598_2020_64726_MOESM1_ESM.pdf]

High resolution behavioral and neural activity representation using a geometrical approach

Zev Brand and Avi Avital

Supplemental Appendix

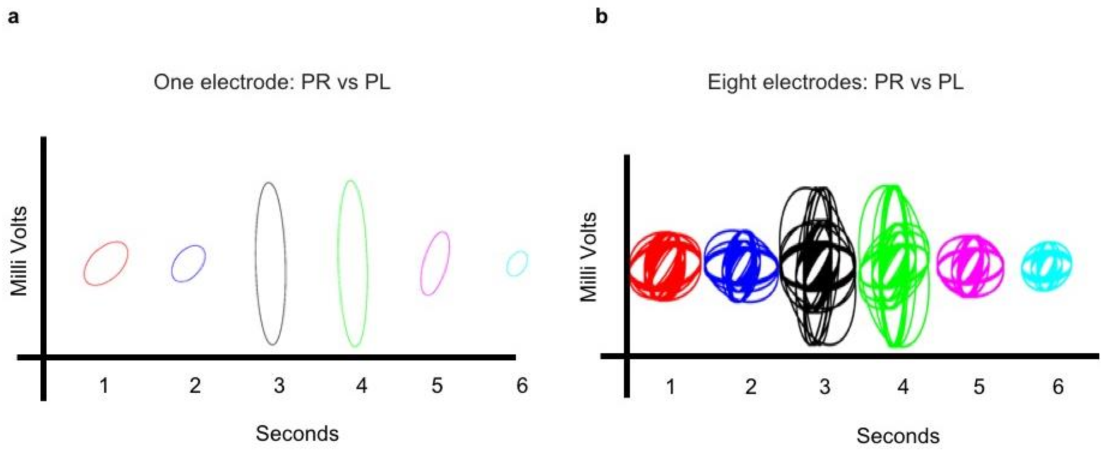

Supplementary Fig 1: Example of synchronization of neural activity (represented by ellipse shape) and behavioral performance. a, Six ellipses of six seconds of recording. Each ellipse represents the covariance between the left vs. right Putamen. **b,** A bi-lateral comparison of covariance between 8X8 of electrodes along 6 seconds from the left vs. right Putamen, yielding 64 ellipses at each time point. PR, putamen right; PL, putamen left.

	PR-PL	PR-MCR	PL-MCL
Before vs During	$P<0.0001$	$P<0.0001$	$P<0.0001$
Before vs After	$P>0.455$	$P>0.97$	$P>0.516$
After vs During	$P<0.0001$	$P<0.0001$	$P<0.0001$

Supplementary Table 1. Post-hoc Tukey analysis comparing feature B during different phases of a task. The analysis was done on all relevant pairs of brain regions. PR, putamen right; PL, putamen left; MCR, motor cortex right; MCL, motor cortex left.

Supplementary Video 1:

Video 1.mp4

Synchronization of the behavioral performance with neural activity recordings at any chosen time point.
